# A global overview of microsporidia infection in HIV/AIDS patients: an updated systematic review and meta-analysis

**DOI:** 10.1093/inthealth/ihaf163

**Published:** 2026-01-28

**Authors:** Ali Asghari, Mohammad Reza Mohammadi, Roumina Norouzi, Shayan Heidari, Farshad Kakian, Ali Pouryousef, Milad Badri, Farajolah Maleki, Maryam Kazem Pour

**Affiliations:** Medical Microbiology Research Center, Qazvin University of Medical Sciences, Qazvin, Iran; Department of Bacteriology, Faculty of Medical Sciences, Tarbiat Modares University, Tehran, Iran; Medical Faculty, University of Belgrade, 11000 Belgrade, Serbia; Medical Faculty, University of Belgrade, 11000 Belgrade, Serbia; Department of Nursing, School of Nursing, Larestan University of Medical Sciences, Larestan, Iran; Leishmaniasis Research Center, Sabzevar University of Medical Sciences, Sabzevar, Iran; Medical Microbiology Research Center, Qazvin University of Medical Sciences, Qazvin, Iran; Zoonotic Diseases Research Center, Ilam University of Medical Sciences, Ilam, Iran; Clinical Research Development Unit, Shahid Mostafa Khomeini Hospital, Ilam University of Medical Sciences, Ilam, Iran; Zoonotic Diseases Research Center, Ilam University of Medical Sciences, Ilam, Iran; Department of Paediatric Dentistry, School of Dentistry, Ilam University of Medical Sciences, Ilam, Iran

**Keywords:** genotype, HIV, microsporidia, prevalence, species, systematic review

## Abstract

**Background:**

This study aimed to update the global estimates of microsporidia prevalence, species/genotype distribution and clinical correlates among human immunodeficiency virus (HIV)/acquired immunodeficiency syndrome (AIDS) patients.

**Methods:**

A systematic search of international databases was conducted for studies published between 1 January 2017 and 11 October 2025. Pooled prevalence and odds ratios (ORs) were calculated using a random effects model with 95% confidence intervals (CIs).

**Results:**

Thirty-nine studies from 18 countries (24 cross-sectional, 15 case–control) were included. The pooled prevalence of microsporidia among HIV/AIDS patients was 12% (95% CI 8.9 to 16), significantly higher than in controls (1.9%; odds ratio 5.7 [95% CI 3.33 to 9.74]). A higher pooled prevalence of microsporidia infection was observed in studies published after 2020 (15.9%), in those with sample sizes ≤100 participants (19.2%), in patients with CD4 counts ≤200 cells/μl (58.4%) and in males (64.9%). The highest country-specific prevalence rates were reported from Malaysia (60.9%), Mexico (48.3%), Malawi (37.0%) and South Africa (32.9%). *Enterocytozoon bieneusi* was the predominant species, accounting for 24 identified genotypes, followed by *Encephalitozoon intestinalis*, *E. cuniculi* and *E. hellem.*

**Conclusions:**

Microsporidia remain a significant opportunistic pathogen in HIV/AIDS patients. Strengthened molecular surveillance, standardized diagnostic protocols and integration of microsporidia screening into HIV/AIDS care are essential to improve outcomes and control transmission.

## Introduction

Microsporidia are diverse unicellular obligate intracellular parasites previously classified as ‘primitive’ protozoa but now recognized as part of the fungi.^1,2^ Phylogenetic studies position microsporidia alongside Cryptomycota as a basal branch of the fungal kingdom.^3^ With >1700 species and 220 genera, these microorganisms are incredibly varied, with 17 species known to infect humans. *Enterocytozoon bieneusi* and *Encephalitozoon* species such as *E. cuniculi*, *E. hellem* and *E. intestinalis*, are the most prevalent microsporidia in both humans and animals.^1,4^

These pathogens have emerged as significant opportunistic agents, particularly in individuals with compromised immune systems, such as those living with human immunodeficiency virus (HIV)/acquired immunodeficiency syndrome (AIDS).^5^ Although first identified in humans in the late 1920s, microsporidia gained prominence in the 1980s with the HIV/AIDS epidemic.^6,7^ The profound immunodeficiency caused by HIV, particularly as it progresses to AIDS, depletes CD4^+^ T cells critical for immune defence, rendering patients vulnerable to opportunistic infections, including microsporidiosis.^8^

In 2023, 39.9 million people globally were living with HIV.^9^ HIV-related causes led to 630 000 deaths, with >60% occurring in Africa.^10^ Despite major advances in antiretroviral therapy (ART), opportunistic infections remain a major cause of morbidity and mortality, especially in low- and middle-income countries where ART access and adherence are limited. These persistent infections underscore the ongoing public health burden associated with advanced HIV disease.^11^

Microsporidial infections are characterized primarily by chronic diarrhoea, malabsorption syndrome, wasting syndrome and other severe gastrointestinal and systemic manifestations that substantially diminish the quality of life and complicate clinical management.^12,13^ These symptoms typically correlate with the degree of immunosuppression, with patients having CD4 T cell counts <100 cells/μl being at the highest risk for severe outcomes.^1,5,8^

This systematic review and meta-analysis aimed to address existing gaps in knowledge regarding the global epidemiology of microsporidiosis among individuals living with HIV/AIDS. By examining the genetic diversity of microsporidia species alongside demographic and clinical characteristics of affected patients, this study provides a comprehensive overview intended to inform future research, clinical management and public health policy. Advancing understanding in this area is crucial for mitigating the persistent health burden of microsporidiosis, particularly among vulnerable populations impacted by HIV/AIDS.

## Methods

### Ethics approval

The current study was approved by the Ethics Committee of Qazvin University of Medical Sciences, Qazvin, Iran (approval no. IR.QUMS.REC.1403.382).

### Study design

This systematic review and meta-analysis followed the Preferred Reporting Items for Systematic Reviews and Meta-Analyses guidelines to ensure methodological rigor and transparency.^14^

### Search strategy

A comprehensive and systematic search of electronic databases, including PubMed, Web of Science, Scopus and Google Scholar was conducted to identify relevant studies published between 1 January 2017 and 11 October 2025. The search terms combined controlled vocabulary (e.g. MeSH terms) and free-text keywords related to ‘microsporidia’, ‘intestinal parasites’, ‘HIV’, ‘AIDS’, ‘prevalence’, ‘species’, ‘clinical symptoms’ and ‘CD4 T cell’. Boolean operators (AND, OR) and truncation were applied to maximize the sensitivity and specificity of the search. Reference lists of included studies and relevant reviews were manually screened to identify additional eligible studies.

### Inclusion and exclusion criteria

Studies were included if they evaluated HIV/AIDS patients/case group or non-HIV/AIDS patients/control group with confirmed microsporidia infection via molecular, microscopic or serological methods; reported prevalence, species/genotype distribution, demographic and clinical characteristics of affected patients and/or CD4 T cell counts; employed a cross-sectional, case–control or cohort design; and were published in English or widely translated languages. Exclusion criteria encompassed studies lacking sufficient data for extraction, case reports, reviews and those involving animal models or *in vitro* settings.

### Data extraction and quality assessment

Two independent reviewers screened titles, abstracts and full texts for eligibility using a pre-defined screening tool. Discrepancies were resolved through discussion or consultation with a third reviewer. Data extracted included study characteristics (author, year, country, design), diagnostic methods, prevalence rates, microsporidia species/genotypes, age, sex, diarrhoea status and CD4 T cell counts. The quality of included studies was assessed using the Joanna Briggs Institute (JBI) Critical Appraisal Checklist. JBI scores categorized studies as low (≤3), moderate (4–6) or high quality (≥7).

### Data synthesis and sensitivity analysis

The meta-analysis was conducted using Comprehensive Meta-Analysis version 3 software (Biostat, Englewood, NJ, USA) considering p-values <0.05 as statistically significant. Pooled prevalence estimates with 95% confidence intervals (CIs) were calculated using a random effects model to account for heterogeneity.^15^ Subgroup analyses were performed based on geographic regions (countries, continents and World Health Organization [WHO] regions), publication years and sample sizes. The pooled random effects odds ratio (OR) was calculated from case–control studies to assess the association between microsporidia prevalence and HIV/AIDS patients. True subgroup meta-analyses for age, sex, CD4 T cell count and diarrhoea status were infeasible due to incomplete denominator reporting across studies. Therefore, pooled proportions were calculated using the distribution of positive microsporidia cases within each category. A forest plot showed the pooled prevalence and 95% CIs, while a funnel plot assessed publication bias. Heterogeneity was quantified using the I^2^ statistic, with thresholds of 25%, 50% and 75% indicating low, moderate and high heterogeneity, respectively. A sensitivity analysis was conducted by excluding studies to assess the robustness of pooled estimates.

## Results

### Study selection

**Figure 1.**
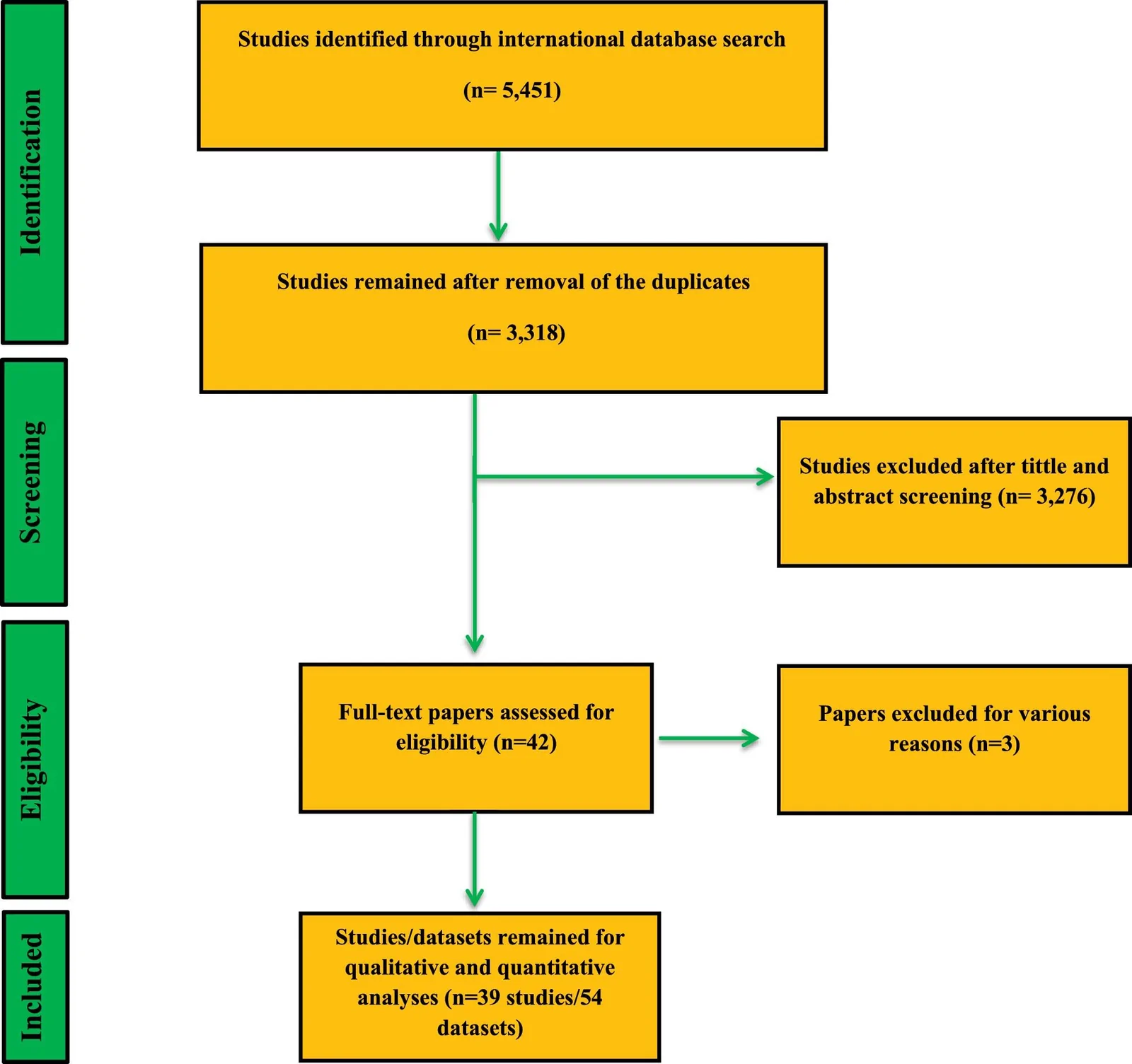
Flow chart illustrating the process of included studies in this systematic review.

The initial search across various databases identified a total of 5451 records. After removing duplicates (2133), 3318 titles and abstracts were screened for relevance. A total of 42 full-text articles were assessed for eligibility, of which 39 met the inclusion criteria (Figure 1).^5,16–53^

### Characteristics of included studies

The included studies, published between 1 January 2017 and 11 October 2025, comprised 24 cross-sectional and 15 case–control studies. Geographically, studies were conducted in 18 countries, with most originating from Iran and India. Sample sizes ranged from 2 to 891 HIV/AIDS patients and diagnostic methods included molecular and microscopic approaches (Table 1).

**Table 1.** Data from 39 articles/54 datasets on the prevalence and genotype distribution of microsporidia infection in HIV/AIDS patients.

				Cases		Controls				
Author, year	Time tested	Country	Study type	Total, N	Infected, n (%)	Total, N	Infected, n (%)	Isolated species	Isolated genotypes	Method
Aissa et al., 2017^16^	2005–2013	Tunisia	CS	108	17 (15.7)	–	–	*E. intestinalis*, *E. bieneusi*	–	MIC, MOL
Kaushik et al., 2017^38^	2014–2016	India	CC	60	8 (13.3)	60	2 (3.3)	*E. bieneusi*, microsporidia spp.	–	MIC, MOL
Khanduja et al., 2017^34^	2010–2014	India	CC	222	4 (1.8)	220	0 (0)	*E. bieneusi*	Ind1, Ind2, Ind3, Ind4	MIC, MOL
Liu et al., 2017^52^	2013–2014	China	CC	285	33 (11.6)	303	0 (0)	*E. bieneusi*	D, IV/K, PigEBITS7, EbpC, GX25, GX456, GX458	MOL
Nooshadokht et al., 2017^30^	UC	Iran	CC	72	10 (13.9)	68	1 (1.5)	*E. bieneusi*	–	MIC, MOL
Tavalla et al., 2017^23^	UC	Iran	CS	102	37 (44.1)	–	–	*E. bieneusi*, *E. intestinalis*, *E. cuniculi*, *E. hellem*	D, M/-/II/1A	MIC, MOL
Tay et al., 2017^33^	UC	Ghana	CC	341	3 (0.9)	331	0 (0)	Microsporidia spp.	–	MIC
Greigert et al., 2018^32^	2014–2016	France	CS	39	2 (5.1)	–	–	*E. bieneusi*	A, D	MIC, MOL
Gupta et al., 2018^53^	2013–2014	India	CS	100	3 (3)	–	–	Microsporidia spp.	–	MIC
Huibers et al., 2018^49^	2010–2012	Malawi	CS	35	13 (37)	–	–	*E. bieneusi*	–	MOL
Sahu et al., 2018^36^	2016–2017	India	CS	105	2 (1.9)	–	–	Microsporidia spp.	–	MIC
Masoumi-Asl et al., 2019^25^	2016	Iran	CS	102	2 (2)	–	–	*E. bieneusi*	–	MIC, MOL
Rodriguez-Perez, 2019^19^	2014–2015	Mexico	CS	29	14 (48.3)	–	–	Microsporidia spp.	–	MIC
Ubanwa et al., 2019^18^	UC	Nigeria	CC	518	33 (6.4)	176	9 (5.1)	Microsporidia spp.	–	MIC
Udeh et al., 2019^24^	2018	Nigeria	CC	891	6 (0.7)	150	0 (0)	*E. bieneusi*	–	MIC
Velásquez, 2019^39^	2012–2018	Argentina	CS	143	18 (12.6)	–	–	*E. bieneusi, E. intestinalis*	–	MIC, MOL
Alain et al., 2020^26^	UC	Cameroon	CC	200	23 (11.5)	100	2 (2)	Microsporidia spp.	–	MIC
de Armas et al., 2020^48^	1995–2008	Spain	CS	514	62 (12.1)	–	–	*E. cuniculi*	–	MIC, MOL
Hosseini et al., 2021^50^	2018–2019	Iran	CS	80	19 (23.7)	–	–	*Encephalitozoon* spp., Microsporidia spp.	–	MIC, MOL
Messaoud et al., 2021^20^	2014–2016	Tunisia	CS	73	12 (16.4)	-	-	*E. bieneusi*	HNM-III, WL12, WL12-like, Peru8, A, B, S5, D	MIC, MOL
Moniot et al., 2021^17^	2017–2019	France	CC	24	5 (20.8)	187	3 (1.6)	*E. bieneusi*	C, IV	MIC, MOL
Samie et al., 2021^35^	UC	South Africa	CS	170	56 (32.9)	–	–	Microsporidia spp.	–	MOL
Khabisi et al., 2022^29^	UC	Iran	CS	50	12 (24)	–	–	*Encephalitozoon* spp., *E. bieneusi*	–	MIC, MOL
Khositharattanakool and Somwang, 2022^28^	2017–2018	Thailand	CS	224	5 (2.2)	–	–	*E. bieneusi*	SH8	MIC, MOL
Al-Brhami, 2022^5^	2019–2020	Yemen	CS	402	57 (14.2)	–	–	Microsporidia spp.	–	MIC
Zhao et al., 2022^51^	2016–2017	China	CC	384	56 (14.6)	199	2 (1)	*E. bieneusi*	–	MOL
Ahmadi et al., 2023^22^	UC	Iran	CC	50	10 (20)	50	3 (6)	*E. bieneusi*, *Encephalitozoon* spp.	D, M	MIC, MOL
Chozas et al., 2023^41^	UC	Spain	CS	81	6 (7.4)	–	–	*E. bieneusi*, *E. intestinalis*	A	MOL
Jiang et al., 2023^31^	2013–2017	China	CS	155	8 (5.2)	–	–	*E. bieneusi*	D, EbpC, IV, Peru11, A, EbpD, I	MOL
Kumar et al., 2023^27^	2013–2017	India	CC	98	17 (17.3)	165	0 (0)	Microsporidia spp.	–	MIC, MOL
Didarlu et al., 2024^37^	2016–2017	Iran	CS	100	18 (18)	–	–	*E. bieneusi*, *E. cuniculi*	–	MIC, MOL
Gómez-Romano et al., 2024^21^	2017–2022	Spain	CS	2	0 (0)	–	–	Microsporidia spp.	–	MOL
Yusoff et al., 2024^40^	2020–2021	Malaysia	CS	23	14 (60.9)	–	–	*E. bieneusi*	–	MIC, MOL
Frickmann et al., 2024^47^	UC	Ghana	CC	595	63 (10.6)	82	0 (0)	Microsporidia spp.	–	MOL
Ramezanzadeh et al., 2024^42^	2019–2021	Iran	CS	137	12 (8.8)	–	–	Microsporidia spp.	–	MIC
Bahrami et al., 2025^46^	2019–2021	Iran	CS	161	8 (5)	–	–	Microsporidia spp.	–	MIC
Nyamngee et al., 2025^43^	2022–2023	Nigeria	CC	750	318 (42.4)	375	72 (19.2)	Microsporidia spp.	–	MIC
Pazmino-Gomez, 2025^44^	2021–2022	Ecuador	CS	85	16 (18.8)	–	–	*E. bieneusi*, *E. intestinalis*	–	MIC
Yurektuk et al., 2025^45^	UC	Turkey	CC	100	17 (17)	50	3 (6)	Microsporidia spp.	–	MOL

UC: unclear; CS: cross-sectional; CC: case–control; MIC: microscopic detection; MOL: molecular detection.

Studies with both case and control groups were treated as two datasets.

### Pooled prevalence of microsporidia in HIV/AIDS patients

**Figure 2.**
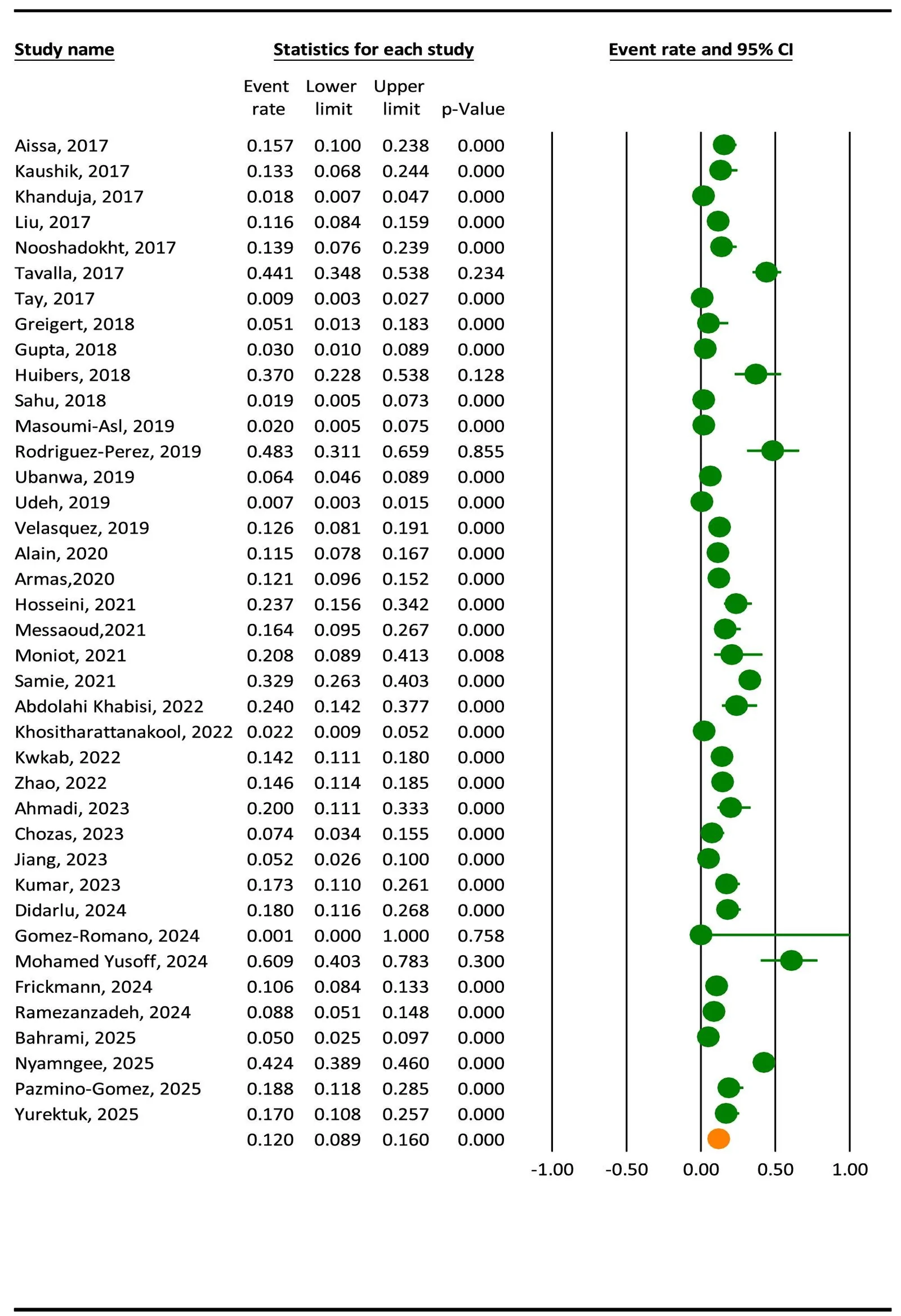
The pooled prevalence of microsporidia infection in HIV/AIDS patients using a random effects model and 95% CIs.

The pooled prevalence of microsporidia infection among HIV/AIDS patients was 12% (95% CI 8.9 to 16), with significant heterogeneity across studies (I^2^=94.7%, p<0.05) (Figure 2).

### Pooled prevalence of microsporidia in non-HIV/AIDS controls

**Figure 3.**
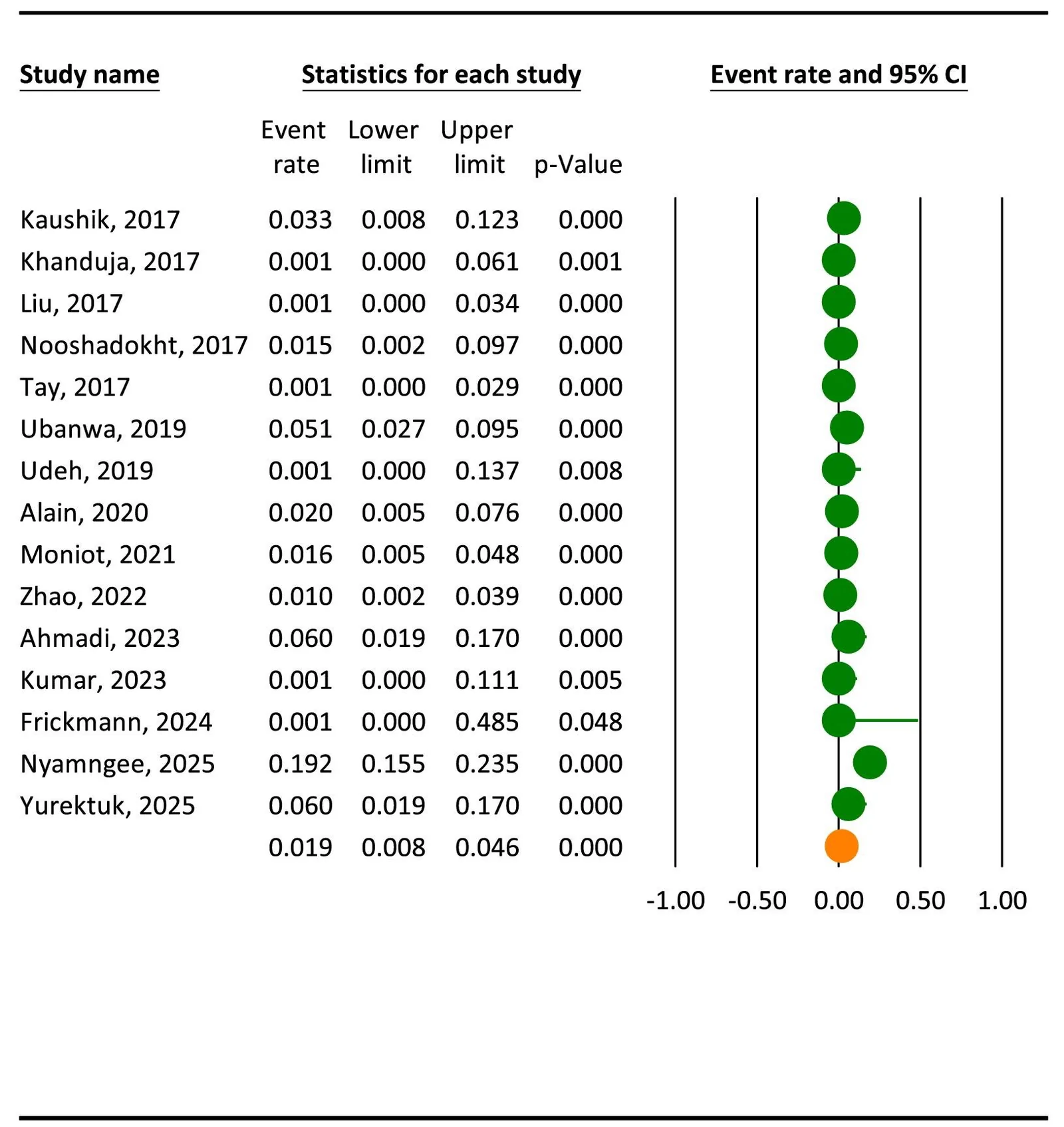
The pooled prevalence of microsporidia infection in non-HIV/AIDS controls using a random effects model and 95% CIs.

The weighted prevalence of microsporidia infection in controls was 1.9% (95% CI 0.8–4.6), showing substantial heterogeneity among studies (I^2^=86.1%, p<0.05) (Figure 3).

### Association between microsporidia infection and HIV/AIDS patients

**Figure 4.**
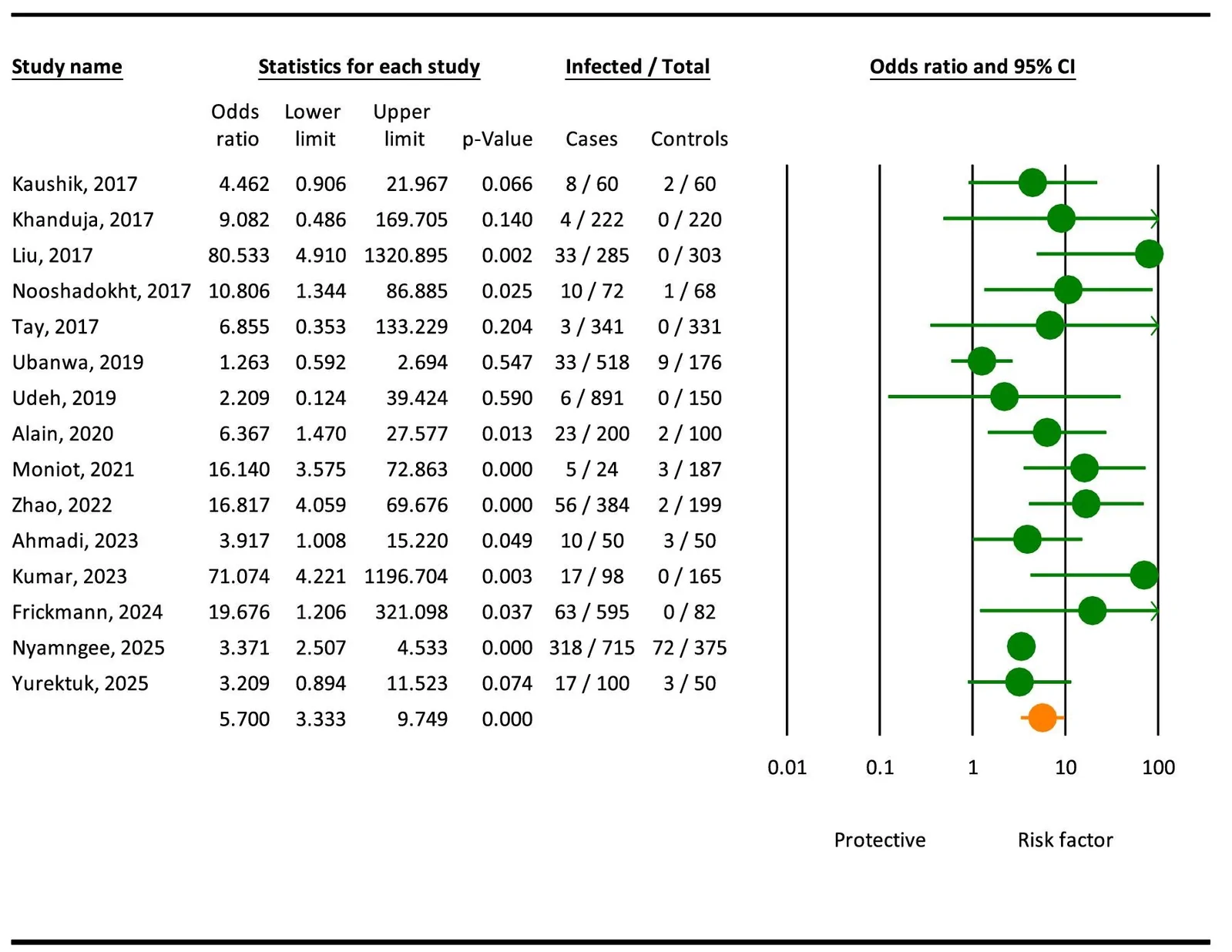
The pooled random effects OR for microsporidia infections in HIV/AIDS patients versus non-HIV/AIDS controls.

The pooled random effects OR for microsporidia infections in HIV/AIDS patients versus controls was 5.7 (95% CI 3.33 to 9.74; p<0.05) (Figure 4).

### Subgroup analysis

Subgroup analyses were performed to explore potential sources of heterogeneity in the pooled prevalence of microsporidia infection among HIV/AIDS patients, based on publication year, continent, WHO region, country and sample size (Table 2).

**Table 2.** Subgroup analysis of microsporidia infection in HIV/AIDS patients by publication year, continent, WHO region, country and sample size.

Subgroup variable	Prevalence, % (95% CI)	Heterogeneity (Q)	df (Q)	I^2^ (%)	p-Value
Publication year					
≤2020	8.4 (5.2–13.4)	253.4	17	93.3	<0.05
>2020	15.9 (11.2–22.3)	362.4	20	94.5	<0.05
Continent					
Africa	11.4 (6–20.7)	437.7	10	97.7	<0.05
Asia	11.4 (7.8–16.4)	189.1	19	89.9	<0.05
Central America	48.3 (31.1–65.9)	0	0	0	>0.05
Europe	11.3 (7.9–15.9)	4.9	4	18.9	>0.05
South America	15.3 (10.2–22.3)	1.6	1	37.4	>0.05
WHO region					
AFR	10 (4.2–22.1)	410.9	7	98.3	<0.05
AMR	23.3 (10.2–45)	17.4	2	88.5	<0.05
EMR	15.4 (10.5–21.9)	83.7	11	86.9	<0.05
EUR	12.6 (9.2–16.9)	7.1	5	29.5	>0.05
SEAR	4.6 (1.7–11.4)	38.7	5	87.1	<0.05
WPR	16.6 (7.9–31.5)	38.4	3	92.2	<0.05
Country					
Argentina	12.6 (8.1–19.1)	0	0	0	>0.05
Cameroon	11.5 (7.8–16.7)	0	0	0	>0.05
China	10.6 (6.7–16.3)	8.7	2	77.1	<0.05
Ecuador	18.8 (11.8–28.5)	0	0	0	>0.05
France	11.5 (2.7–37.8)	3.2	1	68.9	>0.05
Ghana	3.4 (0.3–30.1)	19.1	1	94.8	<0.05
India	5.3 (1.9–14)	29.5	4	86.4	<0.05
Iran	15 (8.6–24.8)	77.3	8	89.6	<0.05
Malawi	37 (22.8–53.8)	0	0	0	>0.05
Malaysia	60.9 (40.3–78.3)	0	0	0	>0.05
Mexico	48.3 (31.1–65.9)	0	0	0	>0.05
Nigeria	6.8 (0.7–42.2)	261.4	2	99.2	<0.05
South Africa	32.9 (26.3–40.3)	0	0	0	>0.05
Spain	11.6 (9.3–14.4)	1.5	2	0	>0.05
Thailand	2.2 (0.9–5.2)	0	0	0	>0.05
Tunisia	16 (11.3–22.1)	0.01	1	0	>0.05
Turkey	17 (10.8–25.7)	0	0	0	>0.05
Yemen	14.2 (11.1–18)	0	0	0	>0.05
Sample size					
≤100	19.2 (14.5–25)	67.7	17	74.9	>0.05
>100	8.2 (5.2–12.7)	644.3	20	96.9	<0.05

By publication period, studies published after 2020 demonstrated a higher pooled prevalence of microsporidia infection (15.9% [95% CI 11.2 to 22.3]) compared with those published in or before 2020 (8.4% [95% CI 5.2 to 13.4]), indicating a possible increase in reported infections in recent years (Supplementary Figure 1).

Across continents, the highest prevalence was observed in Central America (48.3% [95% CI 31.1 to 65.9]), followed by South America (15.3% [95% CI 10.2 to 22.3]), Africa (11.4% [95% CI 6 to 20.7]) and Asia (11.4% [95% CI 7.8 to 16.4]). The lowest prevalence estimates were recorded in Europe (11.3% [95% CI 7.9 to 15.9]). Substantial heterogeneity (I^2^>89%) was noted for Africa and Asia (p<0.05) (Supplementary Figure 2).

When stratified by WHO region, the highest pooled prevalence was observed in the Region of the Americas (AMR; 23.3% [95% CI 10.2 to 45%]) and the Western Pacific Region (WPR; 16.6% [95% CI 7.9 to 31.5]), while the lowest was found in the South-East Asia Region (SEAR; 4.6% [95% CI 1.7 to 11.4]). Marked heterogeneity persisted across most WHO regions (I^2^: 86–98%, p<0.05) (Supplementary Figure 3).

**Figure 5.**
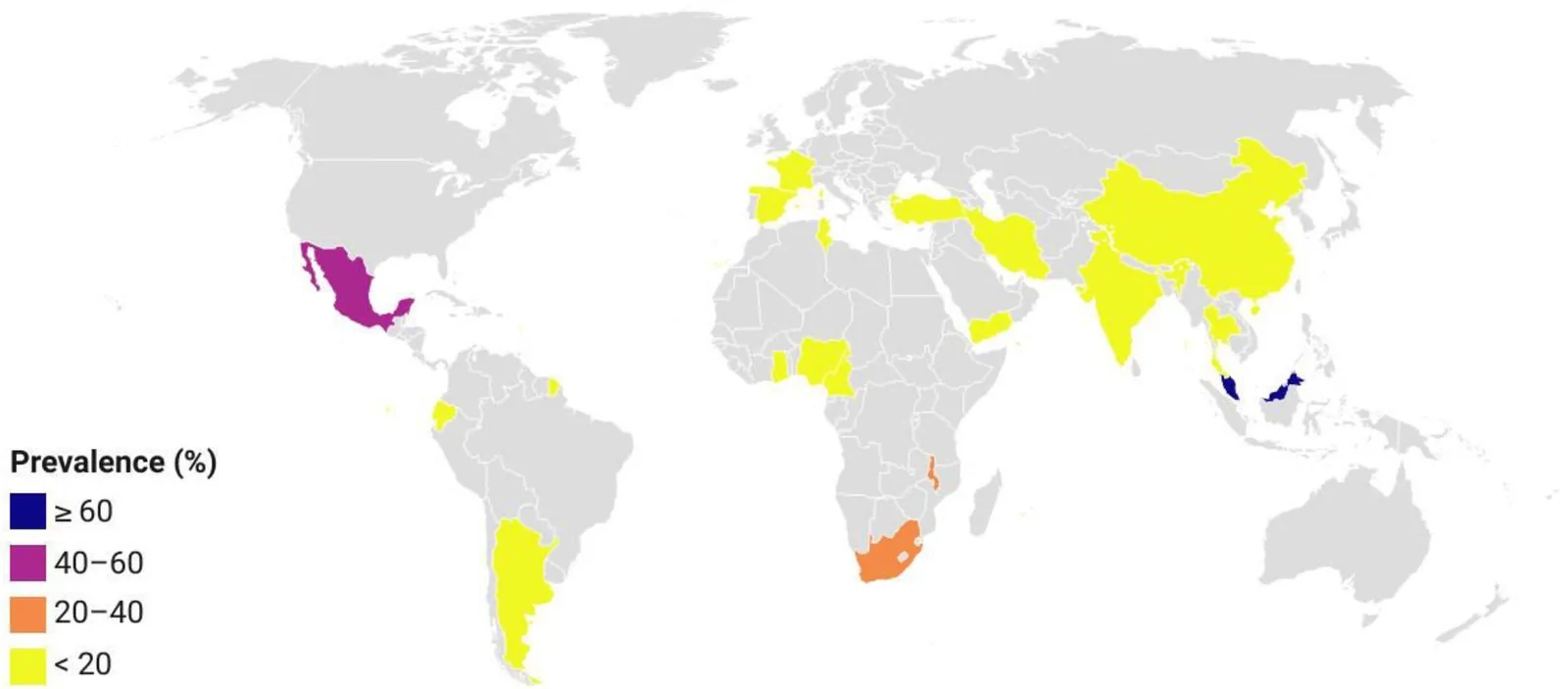
The distribution and prevalence of microsporidia infection in HIV/AIDS patients by country.

Country-specific analyses showed that the highest prevalence was reported in Malaysia (60.9% [95% CI 40.3 to 78.3]), Mexico (48.3% [95% CI 31.1 to 65.9]), Malawi (37.0% [95% CI 22.8 to 53.8]) and South Africa (32.9% [95% CI 26.3 to 40.3]). In contrast, Ghana (3.4% [95% CI 0.3 to 30.1]) and Thailand (2.2% [95% CI 0.9 to 5.2]) showed the lowest prevalence rates (Figure 5). Considerable between-study heterogeneity was evident in several countries, suggesting differences in diagnostic methods, population characteristics or study design (Supplementary Figure 4).

Regarding sample size, studies with ≤100 participants showed a markedly higher pooled prevalence (19.2% [95% CI 14.5 to 25]) compared with studies with >100 participants (8.2% [95% CI 5.2 to 12.7]). Notably, heterogeneity was significantly greater in the latter group (I^2^=96.9%, p<0.05) (Supplementary Figure 5).

### Analysis of demographic and clinical variables

Due to incomplete reporting of total denominators for each variable across studies, subgroup meta-analyses could not be performed for age, sex, CD4 T cell count and diarrhoea status. Instead, pooled proportions were calculated based on the distribution of positive microsporidia cases within each category (Table 3).

**Table 3.** Distribution of positive microsporidia cases in HIV/AIDS patients, categorised by age, sex, CD4 T cell count and diarrhoea.

Variables	Prevalence, % (95% CI)^a^	Datasets^b^, n	df (Q)	Heterogeneity (Q)	I^2^
Age group (years)					
≤40	55.9 (20.8–86)	2	1	3.6	72.5
>40	44.1 (14–79.2)	2	1	3.6	72.5
Sex					
Female	35.1 (20.9–52.5)	4	3	2.4	0
Male	64.9 (47.5–79.1)	4	3	2.4	0
CD4 T cell count					
≤200	58.4 (46.4–69.4)	17	16	38.1	57.9
>200	36.9 (25.3–50.2)	15	14	35.9	61.1
Symptoms					
Diarrhoea	36.4 (27.8–45.9)	7	6	6.1	1.5
Non-diarrhoea	61.9 (49.7–72.8)	5	4	5.8	31.2

a The reported values represent the proportion of microsporidia-positive cases within each category (age, sex, CD4 count and diarrhoea) relative to the total number of positive cases rather than true subgroup-specific prevalence. This is because the denominators (i.e. the total number of individuals tested in each group) were not consistently provided across studies.

b The variation in the number of datasets arises from the fact that not all included studies reported data for every classification within a given variable. For instance, in some studies all HIV/AIDS participants or all microsporidia-positive cases belonged to a single category (e.g. only ≤40 y or >40 y). Such inconsistencies limited the ability to calculate accurate proportions. Therefore, these findings should be interpreted with caution, as they primarily reflect the distribution of available data rather than definitive epidemiological patterns.

Among positive cases, microsporidia infection was more common in males (64.9% [95% CI 47.5 to 79.1]) than in females (35.1% [95% CI 20.9 to 52.5]) (Supplementary Figure 6). Patients ≤40 y of age represented a slightly higher share of positive cases (55.9% [95% CI 20.8 to 86]) compared with those >40 y of age (44.1% [95% CI 14.0 to 79.2]) (Supplementary Figure 7).

Regarding immune status, patients with CD4 counts ≤200 cells/μl accounted for the majority of positive infections (58.4% [95% CI 46.4 to 69.4]) (Supplementary Figure 8). Patients presenting with diarrhoea had a lower pooled proportion of positivity (36.4% [95% CI 27.8 to 45.9]) compared with non-diarrheic individuals (61.9% [95% CI 49.7 to 72.8]) (Supplementary Figure 9), although this finding should be interpreted with caution due to variability in symptom reporting and data limitations.

### Species distribution

Among the studies reporting species data, *E. bieneusi* was the most frequently identified species, followed by *E. intestinalis*, *E. cuniculi* and *E. hellem*. In total, 26 microsporidia genotypes were identified in HIV/AIDS patients, with *E. bieneusi* accounting for the majority (24 genotypes: A, B, C, D, M, I, IV, Ind1, Ind2, Ind3, Ind4, PigEBITS7, GX25, GX456, GX458, HNM-III, WL12, WL12-like, Peru8, S5, SH8, EbpC, Peru11 and EbpD). Several of these genotypes (D, I, EbpC and IV) have been associated with zoonotic transmission. *E. cuniculi* and *E. hellem* each had a single genotype, reported as II and 1A, respectively (Table 1).

### Quality assessment and sensitivity analysis

According to the JBI checklist, 23 studies were rated as high quality and 16 studies as moderate quality (Supplementary Table 1). Sequential exclusion of individual studies did not significantly alter the overall prevalence estimate, indicating the robustness of the findings (Supplementary Figure 10).

### Publication bias

**Figure 6.**
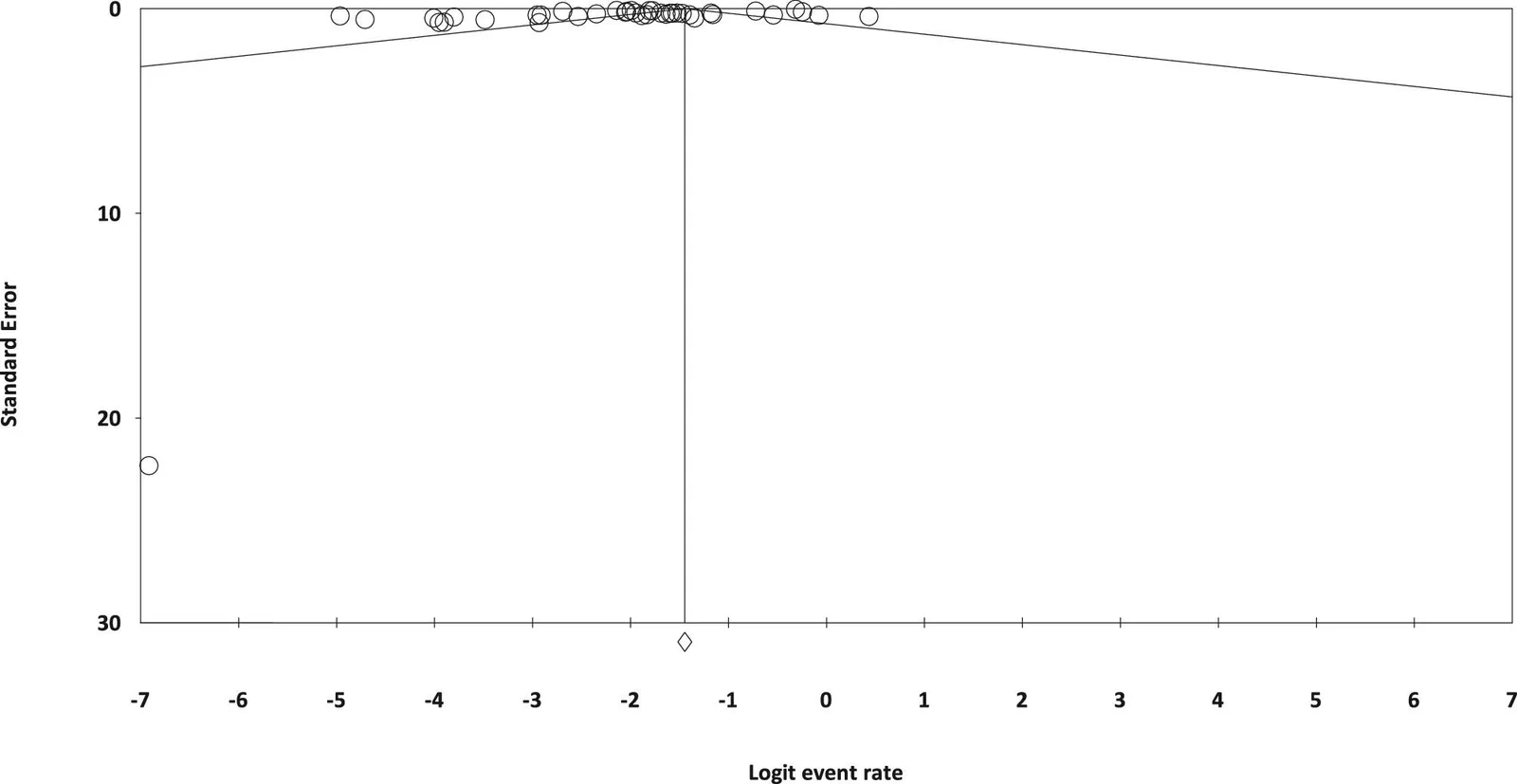
Publication bias in the present study.

The current systematic review and meta-analysis found a significant publication bias (Egger’s regression: intercept=−4.06, 95% lower limit=−6.35, 95% upper limit=−1.77, t-value=3.59, p<0.05) (Figure 6).

## Discussion

Microsporidia are opportunistic intracellular parasites that pose significant health risks to individuals living with HIV/AIDS, particularly those with advanced immunosuppression.^54^ These organisms can cause chronic gastrointestinal and respiratory infections that lead to persistent diarrhoea, weight loss and malnutrition, major challenges in the management of HIV/AIDS.^55,56^ The presence of microsporidia often indicates advanced disease and immune compromise, serving as a marker for other concurrent opportunistic infections.^57–59^ Early diagnosis and treatment are therefore critical in preventing severe complications and improving patient outcomes.^60,61^

Systematic reviews and meta-analyses are essential for clarifying the global epidemiology of opportunistic infections. By synthesizing evidence across regions and populations, they improve statistical power, identify common risk factors and guide both clinical and public health decision-making. The present study updates previous analyses by incorporating recent data (2017–2025) and expanding the scope to include demographic, immunologic and species/genotype-level information on microsporidia infections in HIV/AIDS patients.

A previous meta-analysis of studies up to 2016 found a pooled microsporidia prevalence of 11.8% in HIV/AIDS patients.^57^ In the present updated analysis, which includes 39 studies published between 2017 and 2025, the overall prevalence of microsporidia among HIV/AIDS patients was slightly higher at 12% (95% CI 8.9 to 16). This modest increase suggests that the burden of microsporidia infection remains relatively stable over time, despite the inclusion of recent studies and variations in study design and geographic coverage. The persistent prevalence highlights ongoing diagnostic and management challenges, particularly in resource-limited settings where access to advanced diagnostic tools and antiretroviral therapy may be constrained. Overall, these findings underscore that microsporidia continue to represent a consistent global health concern among people living with HIV/AIDS. The current analysis also confirmed that microsporidia infection was significantly more frequent in HIV/AIDS patients (12%) than in controls (1.9%), with a pooled OR of 5.7 (95% CI 3.33 to 9.74). This strong association highlights the close link between immunodeficiency and susceptibility to microsporidia infection.

Subgroup analysis by sample size revealed that smaller studies tend to report higher prevalence estimates, a pattern often linked to publication bias or the inclusion of high-risk cohorts in small-scale investigations. Consistent with this, the current systematic review and meta-analysis detected significant publication bias. These findings underscore the need for cautious interpretation of prevalence estimates, particularly from smaller studies, and highlight the importance of conducting large-scale, population-based research with standardized diagnostic methods to obtain more reliable and generalizable results.

Notably, the prevalence of microsporidia infection nearly doubled in studies published after 2020. While this trend should be interpreted cautiously, several plausible explanations exist. The apparent increase may reflect disruptions in HIV care and diagnostic services during the COVID-19 pandemic, including delayed ART initiation, reduced access to routine testing or reallocation of laboratory resources away from parasitological diagnostics. Conversely, it could also indicate improved molecular detection techniques or a genuine shift in epidemiology due to changing host or environmental factors. As data remain limited, this observation should be considered exploratory and warrants further investigation through well-designed, post-pandemic surveillance studies.

Geographic analysis revealed substantial variation, with higher prevalence estimates reported from Malaysia (60.9%), Mexico (48.3%), Malawi (37%) and South Africa (32.9%), based on single datasets, while Iran and India contributed a disproportionately large number of studies. This imbalance limits the generalizability of the pooled global estimate. The high prevalence reported in individual countries should be interpreted as preliminary and not necessarily representative of the broader regional situation. Future studies should prioritize underrepresented areas to achieve more balanced and globally representative estimates.

Analyses of demographic and clinical correlates provided additional insights, although they were limited by incomplete data. The higher proportion of infection in younger adults (≤40 y) is consistent with previously reported patterns in HIV epidemiology, where younger individuals often represent the most affected demographic groups.^62^ Among the microsporidia-positive cases, the proportion of HIV/AIDS-positive males was higher than that of females. However, the limited availability of data in this area prevents these findings from being generalized to larger populations. The strong association of microsporidia positivity with CD4 counts ≤200 cells/μl aligns with its well-known role as an opportunistic infection in advanced immunosuppression.^33^ Interestingly, a greater share of microsporidia-positive cases was observed among non-diarrhoeic patients, which may suggest underrecognition of asymptomatic carriage or methodological differences in symptom reporting. However, this observation should be interpreted cautiously, as most studies included were not designed to compare symptomatic versus asymptomatic infections.

Among identified species, *E. bieneusi* was dominant, followed by *E. intestinalis*, *E. cuniculi* and *E. hellem*. A total of 26 genotypes were identified, 24 of which belonged to *E. bieneusi*. The genetic diversity of *E. bieneusi* in HIV/AIDS patients, especially genotypes D, EbpC, IV and I, which are frequently associated with zoonotic transmission, has important public health implications.^63^ These genotypes are known to infect both humans and animals, suggesting multiple transmission routes and potential for cross-species adaptation. Understanding genotype distribution can therefore inform surveillance strategies and highlight the need for a One Health approach to microsporidiosis control.

Therapeutically, albendazole and fumagillin remain the primary agents for treating microsporidiosis; however, their efficacy varies across species and genotypes. Albendazole shows limited activity against *E. bieneusi*, whereas fumagillin is more effective but carries toxicity risks.^56,64^ Furthermore, clinicians should remain vigilant for Immune Reconstitution Inflammatory Syndrome (IRIS) following ART initiation, as immune recovery can paradoxically exacerbate or unmask latent microsporidial infections. Integrating early detection of microsporidia into HIV management protocols may help mitigate such complications.^65,66^

While this updated meta-analysis offers valuable insights into the current epidemiology of microsporidiosis among HIV/AIDS patients, several limitations should be acknowledged. The data were disproportionately derived from a few geographic regions, and some subgroups included only a limited number of studies. The high degree of heterogeneity observed across studies likely reflects differences in diagnostic techniques (e.g. microscopy versus polymerase chain reaction), study populations and regional contexts. In addition, the proportional analyses for variables such as age, sex, CD4 count and diarrhoea were restricted by incomplete data reporting, as the denominators for each variable were often unavailable. As a result, these findings represent proportional distributions among positive cases rather than true subgroup-specific prevalence rates. In addition, evidence of potential publication bias and small-study effects suggest that the pooled estimates should be interpreted with caution. Overall, the findings of the current study lay the groundwork for more standardized and geographically diverse research.

## Conclusions

This updated systematic review and meta-analysis provides an integrated overview of microsporidia infections among HIV/AIDS patients between 2017 and 2025, reaffirming their clinical and epidemiological significance in the era of ART. The pooled global prevalence of 12%, more than fivefold higher than in HIV-negative controls, underscores the continued vulnerability of immunocompromised populations, particularly those with CD4 T cell counts ≤200 cells/μl. The predominance of *E. bieneusi*, with its extensive genotype diversity and zoonotic potential, highlights the importance of adopting a One Health perspective in surveillance and prevention strategies. Marked regional variation and considerable heterogeneity emphasize the urgent need for standardized diagnostic methods, harmonized data reporting and inclusion of underrepresented regions in future research. Clinically, routine screening for microsporidia in HIV/AIDS patients, especially those with advanced immunosuppression or unexplained gastrointestinal symptoms, could improve early detection and management. Strengthening laboratory capacity, integrating molecular diagnostics into routine HIV/AIDS care and promoting multicentric, longitudinal studies will be essential to refine our understanding of transmission dynamics, treatment responses and the broader public health implications of microsporidiosis.

## Supplementary Material

ihaf163_Supplemental_Files

## Data Availability

The datasets used and/or analysed during the current study are available in the online version.
