# Supplementary material for: A global overview of microsporidia infection in HIV/AIDS patients: an updated systematic review and meta-analysis
Source: Int Health. 2026 Jan 28;18(5):705–18. doi: 10.1093/inthealth/ihaf163 (PMC13530294; doi:10.1093/inthealth/ihaf163)
Supplement: Supplementary file 1 [file ihaf163_supplemental_files.zip › Suppl Fig. 6.docx]

**Supplementary Fig. 6.** Pool of positive microsporidia in HIV/AIDS patients by sex.
